# Review of "Concise Clinical Oncology" by Clive Peedell

**DOI:** 10.1186/1477-7800-5-6

**Published:** 2008-02-29

**Authors:** Chung T Lim, Chung S Lim

**Affiliations:** 1 St. Bartholomew and the Royal London School of Medicine and Dentistry, Queen Mary College, University of London, UK; 2 Department of Vascular Surgery, Charing Cross Hospital, Fulham Palace Road, London W6 8RF, UK

## Review

**Figure 1:**
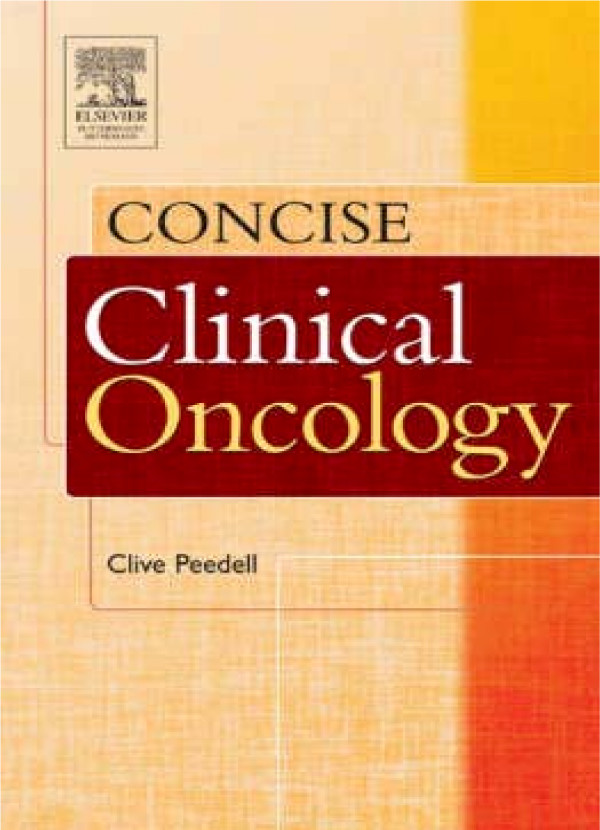
Front Cover of "Concise Clinical Oncology" by Clive Peedell.

"Concise Clinical Oncology" by Clive Peedell is physically portable and handy. Measuring only 188 × 127 mm, it is in the size of any typical medical handbooks available (Figure 1). This makes it a useful pocket-size book, allowing the reader to carry it around conveniently. The design of the book is simple and reader friendly, with well-covered contents and index for easy-referencing.

The author has divided the contents into three main sections. The first section, which consists of ten chapters, explains the general principles of oncology. The second section, which is the main body of the book, discusses the different types of cancers in details. This section is conveniently named "A-Z of cancers". The final section elaborates on the complications and emergencies of cancers (e.g. metastases, malignant pleural effusion, acute bowel obstruction etc.).

Various cancers are discussed in details including their demographic information, pathogenesis, clinical features, management and prognosis. The information provided is concise and yet sufficient for a good understanding of the general principles of oncology and specific cancers. Besides that, the author also discusses various future perspectives and the on-going development in oncology which certainly encourage the reader to explore for new ideas in the challenging world of cancers.

This reader friendly book will be useful and handy to medical students, scientists, clinicians and health-care professionals who have interest in oncology. The medical students will be able to gain a clear understanding of the general principles of oncology and various types of cancers. Meanwhile the clinicians, health-care professionals and scientists may use this book as a quick reference and a reminder of the features of the various cancers.

Finally, we would also recommend each oncology ward to have a copy of this book in the desk especially for its concise and yet invaluable contents. Furthermore, it will hardly take up much space just like any British National Formulary or Oxford Handbooks.

## Competing interests

The author(s) declare that they have no competing interests.

